# Publisher Correction: Methanol fixation is the method of choice for droplet-based single-cell transcriptomics of neural cells

**DOI:** 10.1038/s42003-023-05022-7

**Published:** 2023-06-13

**Authors:** Ana Gutiérrez-Franco, Franz Ake, Mohamed N. Hassan, Natalie Chaves Cayuela, Loris Mularoni, Mireya Plass

**Affiliations:** 1 Gene Regulation of Cell Identity, Regenerative Medicine Program, Bellvitge Institute for Biomedical Research (IDIBELL), L’Hospitalet del Llobregat, Barcelona, Spain; 2 Program for Advancing Clinical Translation of Regenerative Medicine of Catalonia, P-CMR[C], L’Hospitalet del Llobregat, Barcelona, Spain; 3 Regenerative Medicine Program, Bellvitge Institute for Biomedical Research (IDIBELL), L’Hospitalet del Llobregat, Barcelona, Spain; 4 Center for Networked Biomedical Research on Bioengineering, Biomaterials and Nanomedicine (CIBER-BBN), Madrid, Spain

**Keywords:** Transcriptomics, Gene expression, Next-generation sequencing, Gene expression analysis, Cell type diversity

Correction to: *Communications Biology* 10.1038/s42003-023-04834-x, published online 15 May 2023.

In the original version of this Article, the last two paragraphs of the Discussion were arranged out of order, stating:

“Taking into account the pros and cons of the different methods (Table 2) and the limitations of this study, our comparative analysis indicates that methanol fixation is the best preservation method to perform single-cell transcriptomics analyses on neural cells. Libraries from methanol-fixed cells have similar complexity to that of fresh cells (Fig. 2) and do no present strong biases in gene expression that affect the overall transcriptomic profile of cells (Figs. 5 and 6 and Supplementary Data 4 and 5) or cell composition (Fig. 4 and Supplementary Fig. 6), thus providing the sample with the most similar profile to that of fresh cells.

In this work, we have tested the impact of fixation and preservation methods in hiPSC-derived neuronal and glial cells using a few replicates (2–4) per condition. This amount of replicates is acceptable considering the cost of individual single-cell experiments and current experimental standards. Yet, it limits our ability to fully assess the impact of all possible variables in the quality of the sample. Our results show that not only the method of preservation affects sample composition and gene expression. Other parameters such as the days of preservation, the cell line used, the differentiation experiment, and the batch of beads have an impact on the final single-cell transcriptomes. Yet, this work does not provide an extensive comparison of all of them, which is out of the scope of this project. Therefore, the results provided in this work may be different when working with different cell types, samples, and single-cell technology or using different experimental conditions that the ones used here. Researchers should consider all these factors and optimize individual experiments given that our results demonstrate that sample processing can impact significantly the results of single-cell transcriptomics experiments.”

This section should instead read as:

“In this work, we have tested the impact of fixation and preservation methods in hiPSC-derived neuronal and glial cells using a few replicates (2–4) per condition. This amount of replicates is acceptable considering the cost of individual single-cell experiments and current experimental standards. Yet, it limits our ability to fully assess the impact of all possible variables in the quality of the sample. Our results show that not only the method of preservation affects sample composition and gene expression. Other parameters such as the days of preservation, the cell line used, the differentiation experiment, and the batch of beads have an impact on the final single-cell transcriptomes. Yet, this work does not provide an extensive comparison of all of them, which is out of the scope of this project. Therefore, the results provided in this work may be different when working with different cell types, samples, and single-cell technology or using different experimental conditions that the ones used here. Researchers should consider all these factors and optimize individual experiments given that our results demonstrate that sample processing can impact significantly the results of single-cell transcriptomics experiments.

Taking into account the pros and cons of the different methods (Table 2), and the limitations of this study, our comparative analysis indicates that methanol fixation is the best preservation method to perform single-cell transcriptomics analyses on neural cells. Libraries from methanol-fixed cells have similar complexity to that of fresh cells (Fig. 2) and do no present strong biases in gene expression that affect the overall transcriptomic profile of cells (Figs. 5 and 6 and Supplementary Data 4 and 5) or cell composition (Fig. 4 and Supplementary Fig. 6), thus providing the sample with the most similar profile to that of fresh cells.”

This error has now been corrected in the PDF and HTML versions of the Article.

