# Supplementary material for: Methanol fixation is the method of choice for droplet-based single-cell transcriptomics of neural cells
Source: Commun Biol. 2023 May 15;6:522. doi: 10.1038/s42003-023-04834-x (PMC10185690; doi:10.1038/s42003-023-04834-x)
Supplement: Supplementary file 2 — Supplementary Information [file 42003_2023_4834_MOESM2_ESM.pdf]

Supplementary material

Methanol fixation is the method of choice for droplet-based single-cell transcriptomics of neural cells

Ana Gutiérrez-Franco[1,2†], Franz Ake[1,2†], Mohamed N. Hassan[1,2], Natalie Chaves Cayuela[1,2], Loris Mularoni[2,3] and Mireya Plass[1,2,4*]

[1] Gene Regulation of Cell Identity, Regenerative Medicine Program, Bellvitge Institute for Biomedical Research (IDIBELL), L'Hospitalet del Llobregat, Barcelona, Spain

[2] Program for Advancing Clinical Translation of Regenerative Medicine of Catalonia, P-CMR[C], L'Hospitalet del Llobregat, Barcelona, Spain

[3] Regenerative Medicine Program, Bellvitge Institute for Biomedical Research (IDIBELL), L'Hospitalet del Llobregat, Barcelona, Spain

[4] Center for Networked Biomedical Research on Bioengineering, Biomaterials and Nanomedicine (CIBER-BBN), Madrid, Spain.

[†] These authors contributed equally

* Corresponding author: mplass@idibell.cat

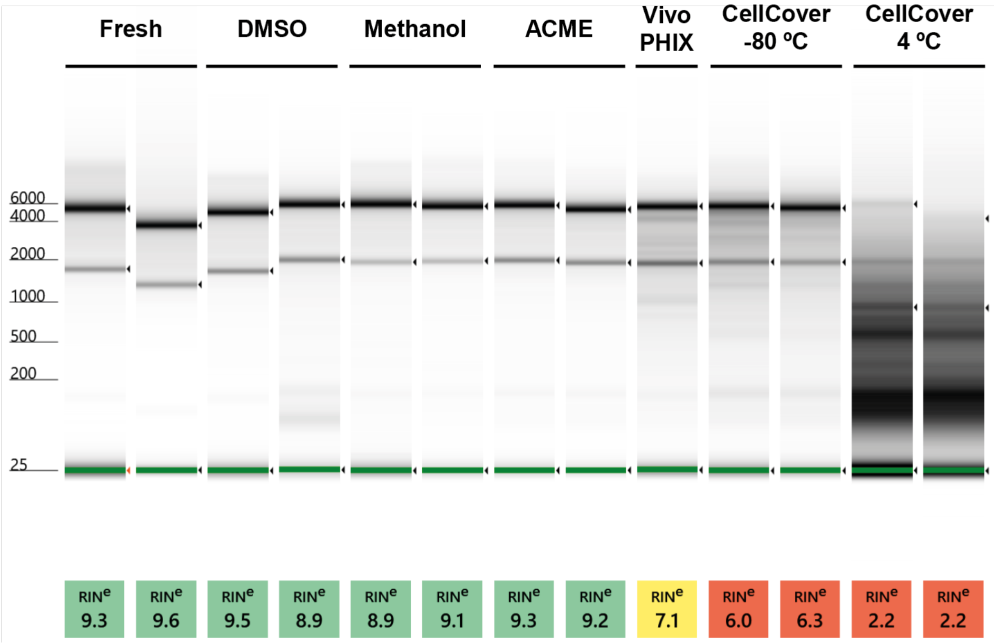

Supplementary Figure 1. RNA quality control of preserved samples. Gel image of the RNA profile of the different samples obtained from a High Sensitivity D5000 ScreenTape assay using a 4200 Agilent Tape Station system. Bands marked with < represent 28S, 18S and the lower marker. The RIN values for each sample is shown below the gel. The RNA quality of DMSO, methanol and ACME samples is very high and equivalent to that of fresh samples. VivoPHIX shows a medium RNA quality while CellCover samples at -80ºC or 4ºC show lower RNA quality, specially at 4ºC.

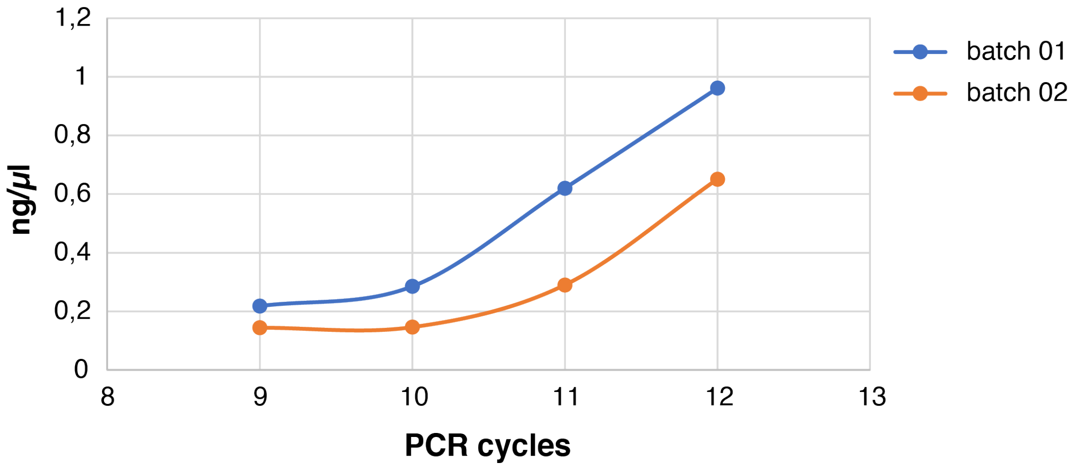

Supplementary Figure 2. Comparison of the capture efficiency of the 2 bead batches used in the project. The plots show the concentration of cDNA in ng µl^{-1} as a function of PCR cycles. Beads from batch 01 have a higher capture efficiency than batch 02, which is reflected in a higher number of genes and UMIs in the samples processed with these beads (Supplementary Table 1).

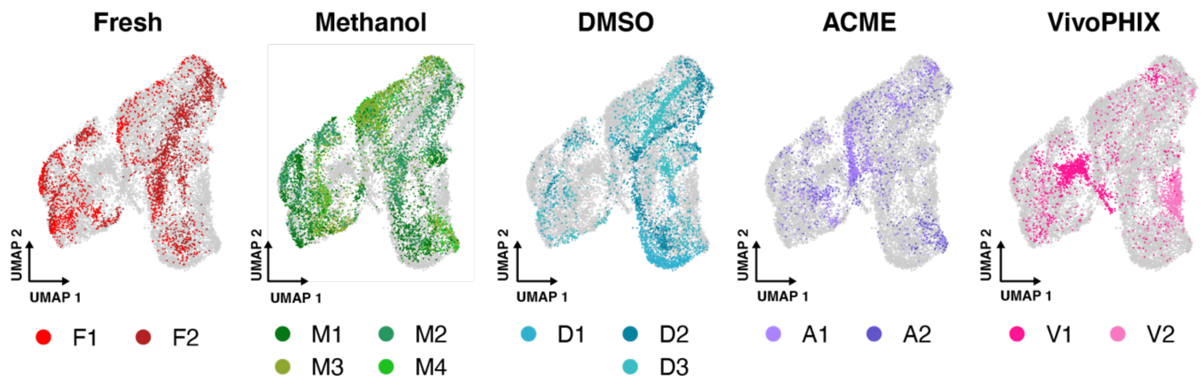

Supplementary Figure 3. Cell preservation induces biases in gene expression that affect sample comparison. UMAP plots showing the distribution of cells from each sample on a UMAP before sample integration. The samples for each processing method (Fresh, Methanol, DMSO, ACME and vivoPHIX) are shown in separate plots. As can be seen from the analyses, cells processed using different methods show different distributions on the UMAP, which highlights the presence of fixation-specific batch effects.

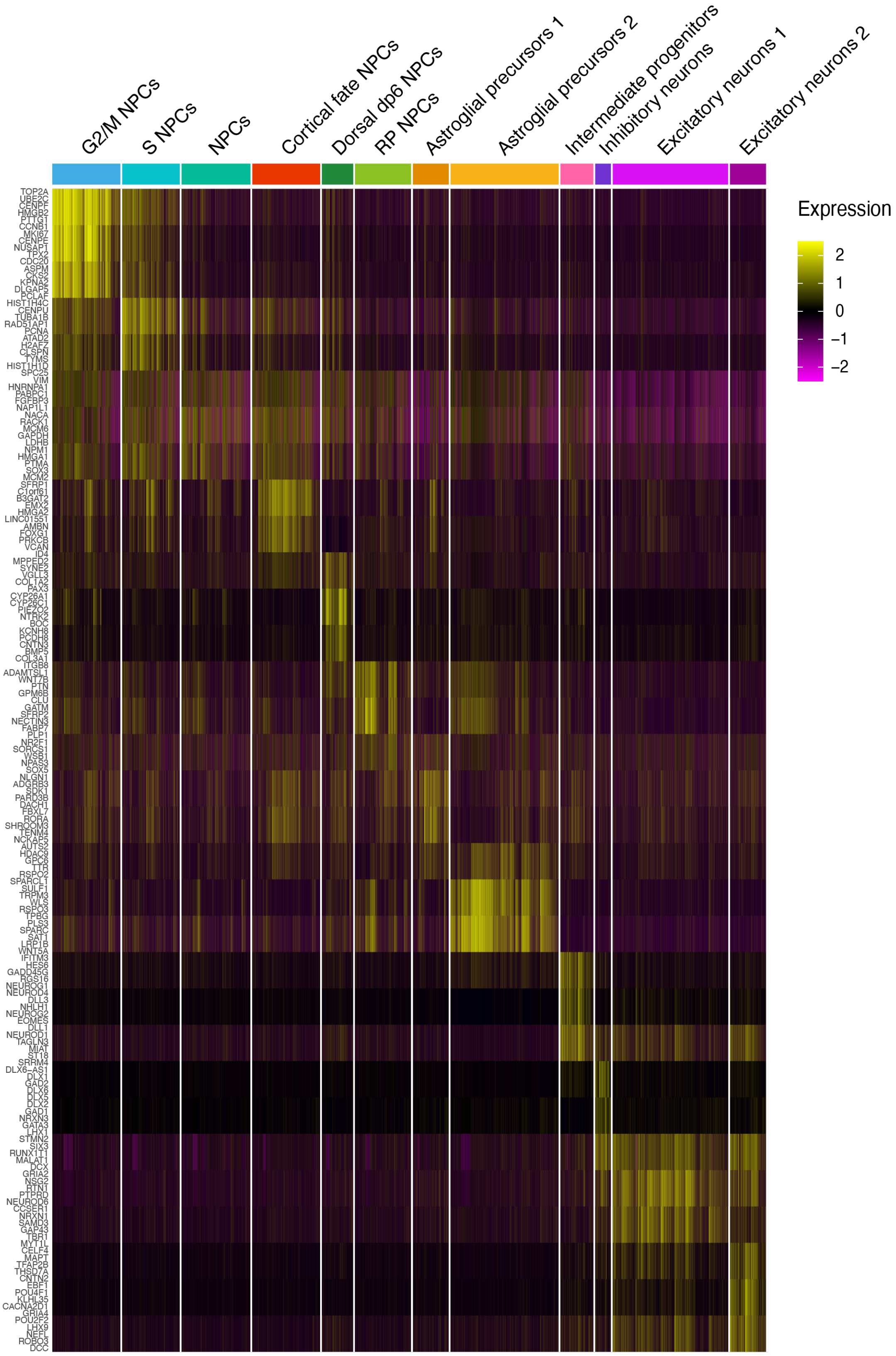

Supplementary Figure 4. Top 15 markers identified for each cluster. Heatmap plot showing the expression of the top 15 markers identified in each cluster in each cell. Normalized expression values for each gene range from purple (low expression) to yellow (high expression).

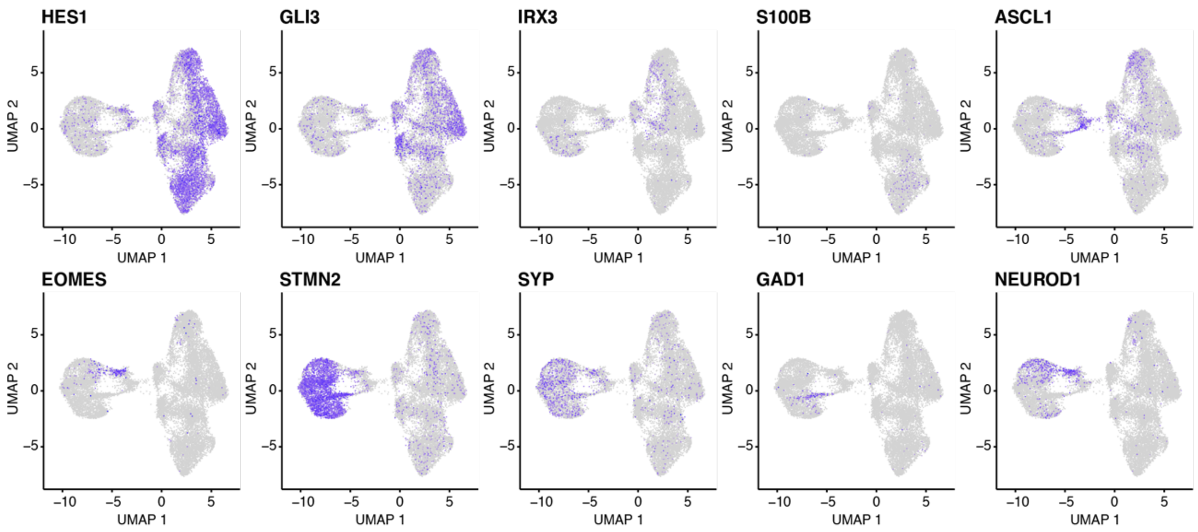

Supplementary Figure 5. Additional feature plots validating the identity of the identified clusters. Feature plots of known markers that have been used to identify the cell populations obtained (Figure 3a). These markers validate the identity of NPCs (*HES1*), cortical fate NPCs (*GLI3*), dorsal fate NPCs (*IRX3*), astrocytes (*S100B*), intermediate progenitor neurons (*ASCL1, EOMES*), immature (*STMN2*) and mature (*SYP*) neurons, and inhibitory (*GAD1*) and excitatory neurons (*NEUROD1*).

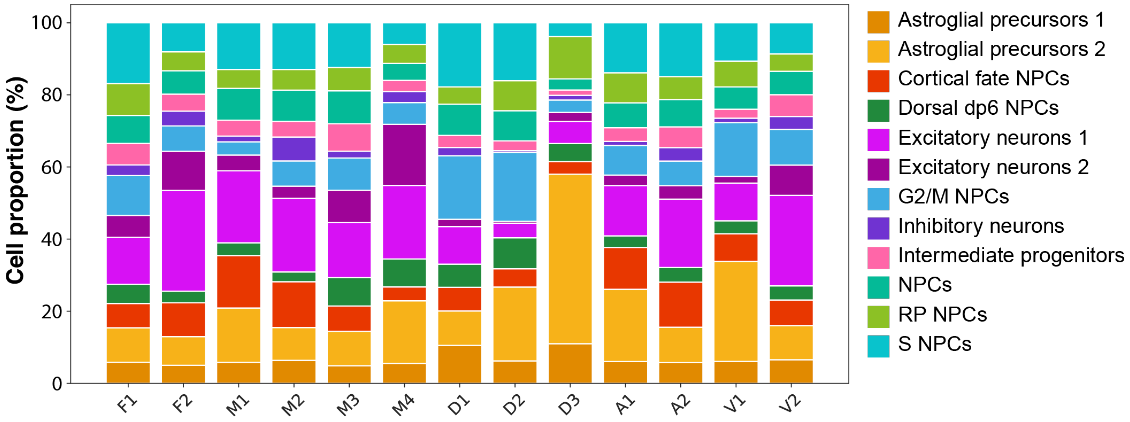

Supplementary Figure 6. Cell composition biases across samples. Stacked barplots showing the proportion of cells in each of the samples. Cell composition changes across samples depends on the cell lines used, the differentiation experiment and the fixation/preservation method used. The analysis with scCODA only identified a significant depletion in excitatory neuron populations in DMSO cryopreserved samples (light and dark purple boxes). The samples are labelled as follows: fresh (F1 and F2), methanol (M1, M2, M3 and M4), DMSO (D1, D2 and D3), ACME (A1 and A2,) and vivoPHIX (V1 and V2).

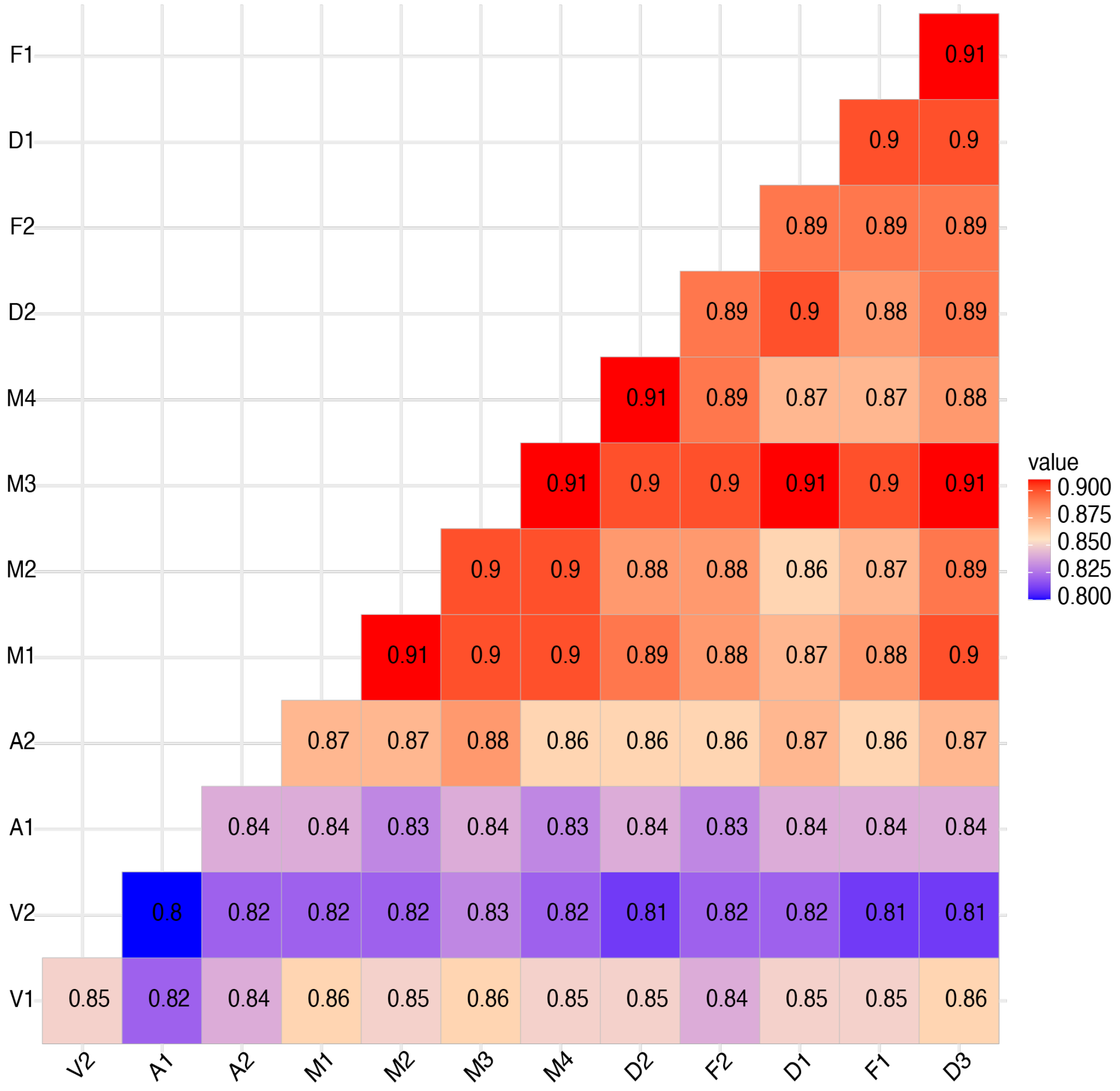

Supplementary Figure 7. Gene expression correlation across samples. Heatmap showing the pseudo-bulk gene expression correlation across samples. Inside each cell, the Pearson correlation coefficient is shown. Both ACME (A1 and A2) and vivoPHIX (V1 and V2) samples show lower correlation with all the other samples. Yet, the average correlation across all samples is quite high (R >= 0.8).

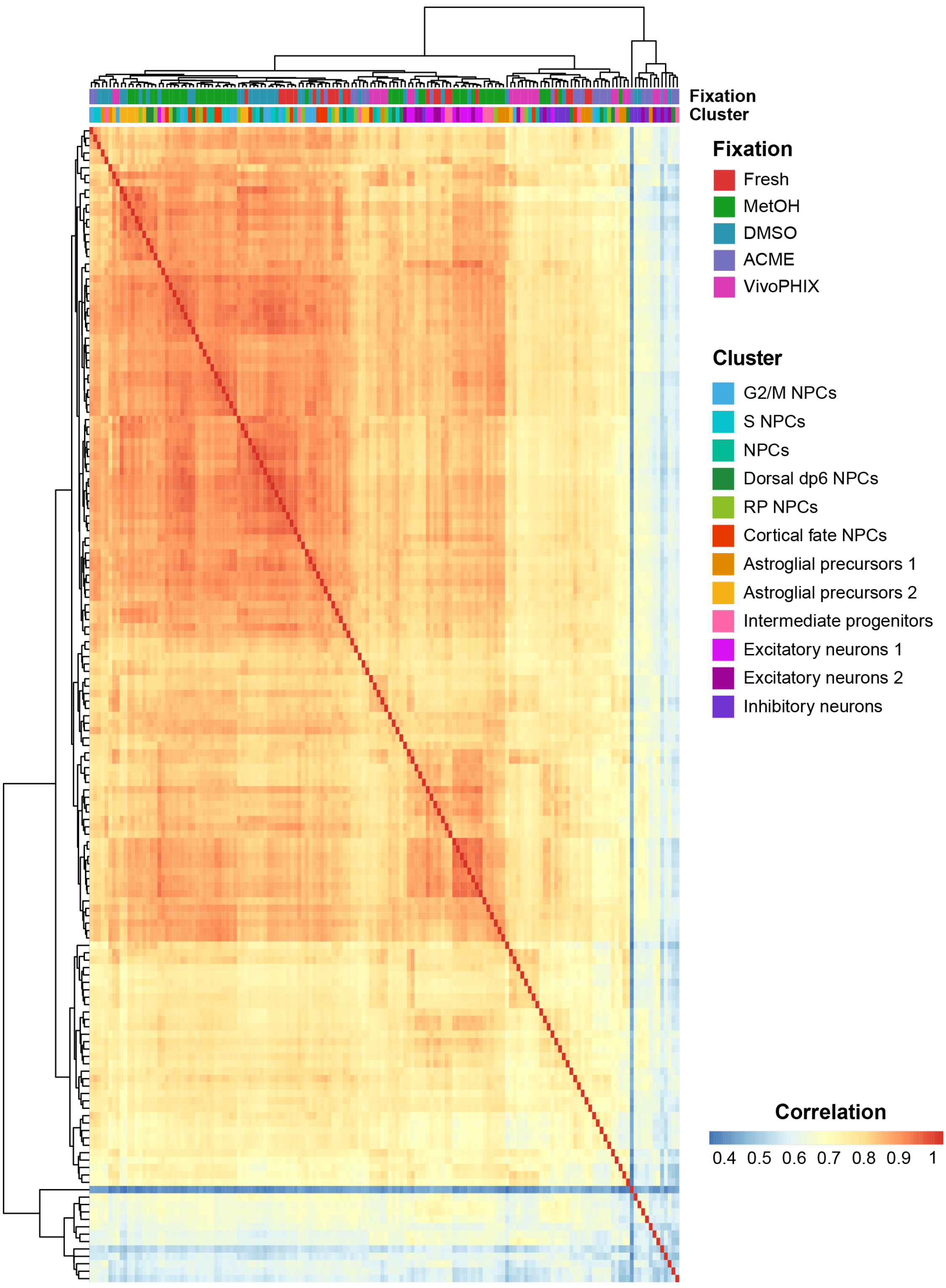

Supplementary Figure 8. Cell-type expression correlation highlights fixation biases in cell clustering. Heatmap plot showing per cluster gene expression correlation across samples. Fixation method and cluster identity for each sample are indicated as color boxes on top of the heatmap. There is an overall good correlation between progenitor cell populations and excitatory neuron populations. Yet, gene expression similarity across clusters does not allow quantifying clustering biases induced by specific fixation methods.

Supplementary References

1. Rodríguez-Traver, E. *et al.* A collection of three integration-free iPSCs derived from old male and female healthy subjects. *Stem Cell Res* **42**, 101663 (2020).
2. Díaz-Guerra, E. *et al.* A collection of four integration-free iPSC lines derived from diagnosed sporadic Alzheimer's disease patients with different APOE alleles. *Stem Cell Res* **39**, (2019).
3. Hutton, S. R. & Pevny, L. H. SOX2 expression levels distinguish between neural progenitor populations of the developing dorsal telencephalon. *Dev Biol* **352**, 40–47 (2011).
4. Yuzwa, S. A. *et al.* Developmental Emergence of Adult Neural Stem Cells as Revealed by Single-Cell Transcriptional Profiling. *Cell Rep* **21**, 3970–3986 (2017).
5. Santos, A., Wernersson, R. & Jensen, L. J. Cyclebase 3.0: A multi-organism database on cell-cycle regulation and phenotypes. *Nucleic Acids Res* **43**, D1140–D1144 (2015).
6. Uzquiano, A. *et al.* Proper acquisition of cell class identity in organoids allows definition of fate specification programs of the human cerebral cortex. *Cell* **185**, 3770-3788.e27 (2022).
7. Wang, H., Ge, G., Uchida, Y., Luu, B. & Ahn, S. Gli3 is Required for Maintenance and Fate Specification of Cortical Progenitors. *Journal of Neuroscience* **31**, 6440–6448 (2011).
8. Legnini, I. *et al.* Spatio-temporal, optogenetic control of gene expression in organoids. *bioRxiv* 2021.09.26.461850 (2022) doi:10.1101/2021.09.26.461850.
9. Hodge, R. D., Kahoud, R. J. & Hevner, R. F. Transcriptional control of glutamatergic differentiation during adult neurogenesis. *Cellular and Molecular Life Sciences* vol. 69 2125–2134 (2012).
10. Karlsson, M. *et al.* A single–cell type transcriptomics map of human tissues. *Sci Adv* **7**, (2021).
