# Supplementary material for: Methanol fixation is the method of choice for droplet-based single-cell transcriptomics of neural cells
Source: Commun Biol. 2023 May 15;6:522. doi: 10.1038/s42003-023-04834-x (PMC10185690; doi:10.1038/s42003-023-04834-x)
Supplement: Supplementary file 3 — Description of Additional Supplementary Files [file 42003_2023_4834_MOESM3_ESM.pdf]

Description of Additional Supplementary Files

File Name: Supplementary Data 1
Description: Overview of samples used in this study

File Name: Supplementary Data 2
Description: Estimated amount of UMI and Genes at 7500 reads per cell

File Name: Supplementary Data 3
Description: Cumulative percentage of cells discarded using each of the different parameters used to remove low quality cells

File Name: Supplementary Data 4
Description: Top-15 markers for the clusters identified

File Name: Supplementary Data 5
Description: Marker genes used to identify cell populations in the scRNA-seq data

File Name: Supplementary Data 6
Description: Differentially expressed genes per cluster for each fixation/preservation method

File Name: Supplementary Data 7
Description: Additional details about the reagents used for cell culture

File Name: Supplementary Data 8
Description: Additional tables with the reagents used for library preparation

File Name: Supplementary Data 9
Description: Parameters and number of doublets identified using DoubletFinder

File Name: Supplementary Data 10
Description: Number of Genes, UMIs, %MT UMIs and % Ribosomal Gene UMIs in fresh and fixed/preserved cells. Source data underlying Figure 2c

File Name: Supplementary Data 11
Description: Number of UMIs assign to intronic or exonic regions in each sample. Source data underlying Figure 2d

File Name: Supplementary Data 12
Description: Percentage of cells assign to each cluster in each individual dataset. Source data underlying Figure 4b

File Name: Supplementary Data 13
Description: Enrichment scores of apoptosis and stress markers in fresh and fixed/preserved samples. Source data underlying Figure 6a & b

File Name: Supplementary Data 14
Description: Number of Differentially Expressed Genes upregulated or downregulated in each cluster for each fixation /preservation method in comparison to fresh samples. Source data underlying Figure 6c
